# Perceived Stress Associated Factors in Workers at a Public University

**DOI:** 10.1590/1980-220X-REEUSP-2022-0219en

**Published:** 2022-11-04

**Authors:** Larissa Bessani Hidalgo Gimenez, Maria Neyrian de Fátima Fernandes, Larissa Horta Esper, Vinicius Santos de Moraes, Ana Carolina Guidorizzi Zanetti, Edilaine Cristina da Silva Gherardi-Donato

**Affiliations:** 1 Universidade de São Paulo, Escola de Enfermagem de Ribeirão Preto, Ribeirão Preto, SP, Brazil.; 2 Universidade Federal do Maranhão, Departamento de Enfermagem, Imperatriz, MA, Brazil.

**Keywords:** Stress, Psychological, Occupational Health, Universities, Estresse Psicológico, Saúde do Trabalhador, Universidades, Estrés Psicológico, Salud Laboral, Universidades

## Abstract

**Objective:**

To describe the predictors of perceived stress in a broad sample of workers at a Brazilian public university.

**Method:**

Cross-sectional study carried out with a convenience sample of workers at a public university in Brazil. To be included in the present study, the worker had to be an administrative technician. From March to August, 2017, workers were surveyed, and 929 participants answered the questionnaires of sociodemographic characterization, work and health conditions, perceived stress (Perceived Stress Scale), depression (Beck Depression Inventory), and anxiety (Beck Anxiety Inventory).

**Results:**

The multiple linear regression showed that higher perceived stress was associated with being younger and male, occupying a higher or technical position, and presenting higher levels of depression and anxiety.

**Conclusion:**

These findings have implications for occupational health nurses and other health professionals to identify workers at risk for chronic and mental illness through predictors of perceived stress and to guide institutions in planning practical actions for stress management interventions.

## RESUMO

**Objetivo:**

descrever os preditores de estresse percebido em uma ampla amostra de trabalhadores de uma universidade pública brasileira.

**Método:**

Estudo transversal realizado com amostra de conveniência de trabalhadores de uma universidade pública no Brasil. Para ser incluído no presente estudo, o trabalhador deveria ser técnico-administrativo. Entre março e agosto de 2017, os trabalhadores foram pesquisados, e 929 participantes no total responderam aos questionários de caracterização sociodemográfica, condições de trabalho e saúde, estresse percebido (Escala de Estresse Percebido), depressão (Inventário de Depressão de Beck) e ansiedade (Inventário de Ansiedade de Beck).

**Resultados:**

A regressão linear múltipla mostrou que maior estresse percebido estava associado a ser mais jovem e do sexo masculino, ocupar cargo de nível superior ou técnico e apresentar níveis mais elevados de depressão e ansiedade.

**Conclusão:**

Esses achados têm implicações para enfermeiros de saúde ocupacional e outros profissionais de saúde identificarem trabalhadores em risco de doenças crônicas e mentais por meio de preditores de estresse percebido e orientar instituições no planejamento de ações práticas para intervenções de manejo do estresse.

## RESUMEN

**Objetivo:**

describir los predictores del estrés percibido en una amplia muestra de trabajadores de una universidad pública brasileña.

**Método:**

Estudio transversal realizado con muestreo de conveniencia de trabajadores de una universidad pública de Brasil. Para ser incluido en el presente estudio, el trabajador debí a ser técnico-administrativo. Entre marzo y agosto de 2017, se encuestó a los trabajadores y 929 participantes en total respondieron los cuestionarios de caracterización sociodemográfica, condiciones de trabajo y de salud, estrés percibido (Escala de Estrés Percibido), depresión (Inventario de Depresión de Beck) y ansiedad (Inventario de Ansiedad de Beck).

**Resultados:**

La regresión lineal múltiple mostró que un mayor estrés percibido se asoció con ser más joven y de sexo masculino, ocupar una posición superior o de nivel técnico y presentar niveles más elevados de depresión y ansiedad.

**Conclusión:**

Estos hallazgos tienen implicaciones para que los enfermeros de salud ocupacional y otros profesionales de la salud identifiquen a los trabajadores en riesgo de enfermedades crónicas y mentales a través de predictores del estrés percibido y guíen a las instituciones en la planificación de acciones prácticas para las intervenciones de manejo del estrés.

## INTRODUCTION

The stress phenomenon is a set of physiological^(1,2)^, psychological^(2)^, and social^(3)^ reactions, unpredictable environmental changes that lead individuals out of balance or homeostasis, triggering a stress response^(4)^. The environmental predictability and physiological limits are key factors shaping the progression of stress responses^(5)^.

From a physiological point of view, there are changes in the body structure and chemical composition characterized by a set of non-specific responses that occur in three possible stages: alarm, resistance, and exhaustion^(1)^. From a psychological perspective, the determination of an event as a stress generator depends on the cognitive evaluation, on the individual’s perception of the situation experienced. Stress responses can progress to pathologies when situational environmental demands outweigh the individual’s perceived psychological and physiological ability to deal with it effectively^(2,6)^. Another approach is understanding stress through a social paradigm, considering the way in which the individual interacts actively with the environment and social events. These can be viewed as potentially threatening or challenging because of their available coping resources^(3)^.

The work environment can be a social place where the individual finds stressors on a daily basis and does not always have the skills or abilities to deal with or modify them^(7)^. High levels of stress in the workplace are associated with damage to the worker’s physical and mental health, with significant repercussions for work productivity and quality of life^(8)^.

Besides working conditions, other predictors, such as socio-cultural aspects, economic factors, and the kind of occupation, might be associated with perceived stress in workers. Studies have shown that workers in educational institutions, especially those with higher education, have high levels of work stress and associations with unhealthy habits^(9,10)^.

The presence of anxious and depressive symptoms seems to be associated with high levels of stress. Stress is among the problems employees confront most often as a broad negative outcome of working life. For this reason, job stress has become a significant social phenomenon and a public health problem^(11)^. In work environments, people may have harmful levels of stress when they do not find enough coping resources to face stressful events^(7)^. Hence, we decided to carry out this study in a group of workers due to their constant complaints on work pressure and the need for the institution to develop a series of programs to increase workers’ well-being.

Administrative technicians in higher education institutions in Brazil might have high perceived stress levels. Therefore, perceived stress is the interaction between individuals and their environment, assessed as threatening or straining their resources in a way that will affect their well-being^(2)^. Estimating perceived stress is one of the measures allowing the understanding of this subject, considering the individualities and specific coping mechanisms. Thus, the purpose of this study is to describe the predictors of perceived stress in a broad sample of workers at a Brazilian public university.

## METHOD

### Design of Study

We conducted a cross-sectional study with administrative technicians in a public university located in the state of Sao Paulo, Brazil. The guidelines suggested by the Strengthening the Reporting of Observational Studies in Epidemiology (STROBE) were followed.

### Local

The public university where the study was carried out is divided in two parts: eight teaching units and four administrative sectors. About the work levels at the institution, administrative technicians occupy different positions at the campus. These workers fell into three divisions, depending on their level of education: 1. elementary level (elementary education); 2. technical level (high school); and 3. higher level (undergraduate).

Professionals of elementary level perform essential general service functions, such as cleaning, janitor, gardener services, among others. Workers in technical level positions have administrative jobs (e.g., secretary, financial technician, laboratory technician). Workers in higher-level positions have coordinating or supervisory roles and are usually responsible for an administrative sector or for a laboratory (e.g., nurses, doctors, chemists, teachers, administrators, etc.). Some positions, achieved through a selection test, are full-time jobs and others are part-time jobs, depending on the work regime at hiring.

### Sample Characteristics

Participants were a convenience sample of administrative technicians from administrative sectors in education from a public university campus formed by a wide range of ages, socioeconomic and educational levels, and job functions. To work in the public sector, the individual does the public service entrance exam and, if approved, acquires stability in public services. In this study’s data collection period, the number of administrative technicians was 1,704. With G*Power software (version 3.1.9.7; Heinrich-Heine-Universität Düsseldorf, Düsseldorf, Germany) using a 5% significance level and a statistical power of 95% in a F test, it was established that a minimum of 280 participants were required. The post hoc for F test in the final sample of 929 showed a statistical power of 99%.

### Selection Criteria

To be included in the present study, the worker had to be an administrative technician.

### Data Collection Procedures

Between March and August 2017, we surveyed participants in their respective workplaces. A data collector from the research team approached each subject during their working time in the university departments. The data collector explained the purpose of the study, and subjects participated in the survey voluntarily. Written consent was obtained from workers who agreed to participate. With some brief instructions, each participant was asked to complete the questionnaires.

**Figure 1.**
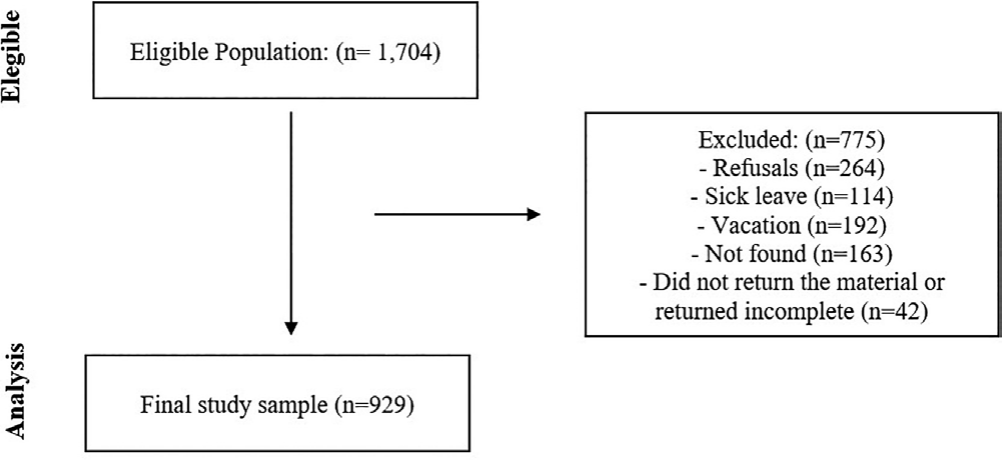
Schematic flow of data collection.

Those that accepted to participate received a closed envelope containing a paper with a self-administered survey. As an effort to address potential sources of bias, the questionnaires were handed out directly to workers and respondents had at least one week to return it to the research team. In addition, the questionnaire’s order was randomly changed in each envelope. It was pointed out that participants could contact the researchers in case of doubt. We excluded incomplete instruments and those who did not respond to one of the questionnaires. The procedure and schematic flow of data collection are described below in Figure 1.

### Measures

The Questionnaire on Sociodemographic, Labor, and Health Conditions had questions related to age, sex, educational level (elementary, high school, undergraduate, or graduate studies), relationship status (single or married), children, job category (elementary, technical, or higher level), time of work (years), side job, religion, physical activity, meditation practice, tobacco use, alcohol use, psychiatric medication.

The Beck Depression Inventory (BDI)^(12)^, validated to Brazilian Portuguese^(13)^, assess depressive symptoms. It consists of 21 statements about depressive symptoms in the last 15 days classified on an ordinal scale from zero to three, producing total scores ranging from zero to 63. A higher score on the scale indicated a higher level of depression symptoms.

The Beck Anxiety Inventory (BAI)^(14)^, validated to Brazilian Portuguese^(15)^, assess symptoms of anxiety. It consists of 21 Likert questions, and the options vary from “absolutely not” to “seriously”. The questions approach how the individual has felt in the last week, expressed in typical anxiety symptoms (such as sweating and feelings of distress). The scores range from zero to 63. A higher score on the scale indicates a higher level of anxiety symptoms.

The Perceived Stress Scale (PSS14)^(3)^ was translated and validated into Brazilian Portuguese^(16)^ and is used to assess the level of perceived stress. This scale is considered a general scale able to assess self-reported stress, which can be used in different age groups as it does not contain context-specific questions. Its reliability was evaluated for the internal consistency in its validation for Brazil, verified through the Cronbach’s Alpha coefficient (α = 0.82). It contains 14 Likert questions, and response options range from zero to four. The questions were designed to assess how unpredictable, uncontrollable, and overloaded respondents evaluate their lives and the “negative perception” and “positive perception” factors. The total score can range from zero (no stress) to 56 (extreme stress). A higher score on the scale indicates a higher level of perceived stress symptoms.

### Statistical Analysis

The completed questionnaires data were analyzed using the Statistical Package for Social Science, version 17.0 (SPSS 17.0, Chicago, IL). The significance level was set at a value of p < 0.05 for all analyses. The studied variables were described in their frequencies, both in absolute and percentage numbers, in their means and standard deviations (SD).

We performed a multiple linear regression in their unadjusted beta and 95% confidence intervals (95% CI) for each variable and an adjusted model. Due to the exploratory nature of this study, all the variables were tested alone; although not reaching the significance criterion ≤0.005, they were hypothesized as relevant for the prediction and understanding of the phenomenon of interest tested in an adjusted model.

### Ethical Aspects

The project met the specifications in accordance with the resolution of the National Health Council (CNS) 466 of 2012, with approval by the Research Ethics Committee of EERP of Universidade de São Paulo under opinion no. 2.104.739/2017. The first phase of the research project was entitled “Mindfulness-based intervention for reducing perceived stress, depression and anxiety among workers in a public university: randomized clinical trial”. All the individuals included in the study informed their willingness to participate at the consent form.

## RESULTS

### Participant Characteristics

A total of 929 volunteers returned the completed data collection material, the response rate corresponding to 54.51% of the total number of administrative technicians from the investigated campus. All the participants included in the final analysis completed the four questionnaires for data collection.

Table 1 presents the characteristics of the sample. The mean age was 46.15 (SD = 10.57) years, with no difference in sex distribution (p = 0.140). In general, the education level is varied (p < 0.001): 2.80% had elementary school level; 23.80%, high school level; 44.80%, undergraduate level; and 28.60%, graduate level. Most of the participants were in a marital relationship (71.04%, p < 0.001). Likewise, most of them described having children (66.20%, p < 0.001) and still being responsible for raising them (82.52%, p < 0.001). Most workers who answered the study had jobs at technical level (70.18%, p < 0.001).

**Table 1.** Sociodemographic, work and clinical characteristics (n = 929) – Ribeirão Preto, SP, Brazil, 2017.

Sample characteristics		P value
Age, years (mean (SD))^1^	46.15 (10.57)	
Men (n (%))	442 (47.58)	p = 0.140
Education level (n (%))		p < 0.001
Elementary	26 (2.8)	
High school	221 (23.79)	
Undergraduate degree	416 (44.78)	
Graduate degree	266 (28.63)	
In a marital relationship (n (%))	660 (71.04)	p < 0.001
Having children (n (%))	615 (66.20)	p < 0.001
Responsible for children (n (%))	510 (82.52)	p < 0.001
Job level (n (%))		p < 0.001
Basic	124 (13.35)	
Technical	652 (70.18)	
Higher	153 (16.47)	
Relation job/education (n (%))		p < 0.001
Below	605 (65.12)	
Correspondent	313 (33.69)	
Above	11 (1.19)	
Time working, years (mean (SD))^2^	18.54 (11.08)	
Other job (n (%))^3^	63 (6.81)	p < 0.001
Practice religion (n (%))	467 (50.27)	p = 0.870
Practice physical exercise (n (%))	519 (55.87)	p < 0.001
Practice meditation (n (%))^4^	191 (20.58)	p < 0.001
Tobacco use (n (%))	78 (8.40)	p < 0.001
Alcohol use (n (%))	453 (48.76)	p = 0.450
Psychiatric medication (n (%))	135 (14.53)	p < 0.001
BDI depression (mean (SD))	9.02 (7.12)	
BAI anxiety (mean (SD))	7.41 (7.52)	
PSS14 perceived stress (mean (SD))	22.71 (9.52)	

Pearson’s qui-square test. Alpha criteria = 0.05. df: degree of freedom; SD: standard deviation; BDI: Beck’s depression inventory (ref); BAI: Beck’s anxiety inventory (ref); PSS14: Perceived Stress Scale (ref). ^1^Missing:1; ^2^Missing:10; ^3^Missing:4; ^4^Missing:1.

As for the correspondence analysis between educational level and the job position held, 65.12% of the workers occupied positions below their educational levels; 33.69% compatible positions, 1.19% positions above their educational levels (p < 0.001).

The time working at the university was 18.54 (SD = 1.08) years. Moreover, only 6.81% of the participants described having a side job (p < 0.001). There was no difference in the distribution among workers with (50.27%) and without any religious practice (p = 0.870).

Regarding health practices and conditions, 55.87% practiced physical activities (p < 0.001), while a smaller percentage of participants described that they practiced some meditation regularly (20.58%, p < 0.001). New contrast was observed about smoking and alcohol consumption, with 8.40% of the participants smoking (p < 0.001) and 48.76% describing regular use of alcoholic beverages. Regarding alcohol consumption, although being a considerable percentage, it was not different from the percentage of those without alcohol consumption (p = 0.450).

The affirmative answer for using some psychotropic medication corresponded to 14.53% (p < 0.001). The mean scores for symptoms of depression and anxiety were, respectively, 9.02 (SD = 7.12) and 7.41 (SD = 7.52). The mean perceived stress score measured by the PSS14 scale was 22.71 (SD = 9.52) points.

### Predictors of Perceived Stress

Table 2 shows the multiple linear regression models not adjusted and adjusted for the perceived stress scores.

**Table 2.** Multiple linear regression model in predicting perceived stress (PSS14) – Ribeirão Preto, SP, Brazil, 2017.

	Unadjusted Beta (95% CI)	p	Adjusted Beta (95% CI)	p
Age, years (mean (SD))	−0.23 (−0.28; −0.17)	**< 0.001**	−0.14 (−0.20; −0.07)	**< 0.001**
Men (n (%))	2.81 (1.59; 4.02)	**< 0.001**	0.88 (−0.003; 1.76)	**< 0.001**
In a marital relationship (n (%))	−1.96 (−3.30; −0.61)	**0.004**	−0.89 (−1.89; 0.11)	0.080
Having children (n (%))	−2.20 (−3.49; −0.91)	**0.001**	0.70 (−0.33; 1.73)	0.185
Tobacco use (n (%))	1.46 (−0.75; 3.67)	0.194	−0.46 (−1.97; 1.05)	0.548
Alcohol use (n (%))	−0.82 (−2.05; 0.41)	0.190	0.08 (−0.78; 0.94)	0.854
Practice religion (n (%))	−1.66 (−2.88; −0.44)	**0.008**	−0.42 (−1.27; 0.43)	0.332
Practice physical exercise (n (%))	−1.84 (−3.07; −0.61)	**0.003**	−0.43 (−1.27; 0.42)	0.324
Practice meditation (n (%))	−1.49 (−3.00; 0.03)	0.055	−0.80 (−1.84; 0.23)	0.127
Time working, years (mean (SD))	−0.18 (−0.24; −0.13)	**< 0.001**	−0.03 (−0.09; 0.03)	0.369
Job level (n (%))^1^				
Basic (ref)	–	–	–	–
Technical	2.45 (0.63; 4.28)	**0.009**	1.60 (0.33; 2.86)	**0.013**
Higher	−0.11 (−0.30; 4.20)	0.089	1.73 (0.19; 3.28)	**0.028**
BDI score (mean (SD))	0.95 (0.89; 1.01)	**< 0.001**	0.68 (0.60; 0.77)	**< 0.001**
BAI score (mean (SD))	0.78 (0,72; 0.85)	**< 0.001**	0.31 (0.24; 0.39)	**< 0.001**

Alpha criteria = 0.05. PSS14: Perceived Stress Scale; CI: confidence interval; SD: standard deviation; ref: reference; BDI: Beck’s depression inventory; BAI: Beck’s anxiety inventory. ^1^Missing: 2.

We observed that the following social variables were associated with a decrease in the stress score: age (p < 0.001); being in a stable affective relationship, e.g. marriage, (p = 0.004); having children (p = 0.001); practicing some religion (p = 0.008); practicing some regular physical activity (p = 0.003); years of work (p < 0.001). Meditation practice did not reach significance as a predictor of perceived stress scores (p = 0.055).

Among the variables tested and associated with increased perceived stress score, we found that the following increased the perceived stress score: being a man, by 2.81 (p < 0.001); occupying a position classified as technical level when compared to the basic level (beta = 2.45, p = 0.009); and, more moderately, depression and anxiety scores (BDI beta = 0.95, p < 0.001, BAI beta = 0.78, p < 0.001). The meditation practice suggests the direction in favor of protection against perceived stress, but its effect did not reach the significance criterion (beta = −1.49, p = 0.055).

Among those variables which were individually associated with the perceived stress score, only five of them remained as predictors in the adjusted model: age (p < 0.001), being male (p < 0.001), occupying a technical level position when compared to those of basic level (p = 0.013), and the depression (p < 0.001) and anxiety (p < 0.001) scores. In the adjusted model, occupying a higher-level position also reached significance, predicting increased perceived stress when compared to the basic level (beta = 1.73, p = 0.028). The adjusted model explained 57.9% of the variation in the data of the perceived stress scale.

## DISCUSSION

The results contribute to understanding the phenomenon of perceived stress in occupational health, particularly in the type of context investigated. The study findings identified that the lower the age, the greater the perceived stress in the worker (in an adjusted model p < 0.001). These data are similar to the results found by another study^(17)^ that reported high levels of perceived stress in young adults when they started their work activities. However, in the current literature, we have not found a study of this nature comparing the perception of stress among workers of different age groups.

Men and women react differently to stress due to biological and psychosocial systems. The neurobiological foundations of this distinction have been explored, and we must also examine the determinants of environmental and social influence in the stress reaction^(18)^. Another study showed that men and women underwent two stress measurements, a psychological (perceived stress) one and a physiological (serum cortisol)^(19)^ one. As a result, we found that both presented similar high levels of perceived stress. However, they demonstrated different physiological stress levels, where men showed a robust response and women delivered a lower response.

The results obtained in this study show that being male is associated with a higher perception of stress, both in the adjusted and unadjusted analysis, which corroborates the importance of investigating perceived stress, anxiety, and depression in other contexts, such as in the social and work settings.

We identified that about 64% of the sample developed professional activities below their educational level. It is known that less educationally qualified employees experience higher levels of occupational stress than the highly educationally qualified^(20)^. On the other hand, evidence indicates that individuals working in jobs that do not match their level of education can experience demotivation, higher levels of stress, low productivity, lower job satisfaction, and conflicts in the work environment^(21)^. This paradox could be related to these professionals’ high levels of perceived stress.

A positive association was also observed between a higher level of professional position and the higher perception of stress compared to the elementary level. In a multicenter study carried out with public workers from six higher education institutions in Brazil, it was found that jobs with high demand and low control were associated with physical consequences to the individual, including a higher propensity to migraine^(22)^.

According to the stress of higher status hypothesis, workers with more significant resources often experience tremendous stress; such status-related concerns are believed to contribute to chronic stress^(23)^. The results of this study are relevant because high stress in the higher-level professionals represents the presence of stress in the leadership, which can have important consequences to the work environment in terms of communication and social support for the team.

We did not identify the association between alcohol and tobacco use and the perception of stress level. In contrast to this finding, a study with the same sample profile identified a positive association between occupational stress and alcohol use^(10)^.

Evidence indicates that individuals tend to self-medicate psychological stress with alcohol as a coping strategy^(24)^. Within the cause-effect perspective, in the face of exposure to stress at work, alcohol develops a mediating action due to its effects (stimulant and sedative). However, recent research discusses the complexity of this relationship and considers a multidirectional model to understand the problem better, including intervening variables, multiple moderating variables, gender, and different patterns of alcohol use^(24)^. Future research could consist of complex models for this same analysis and use validated instruments to assess alcohol and tobacco use.

Finally, the results indicate an association between perceived stress and symptoms of anxiety and depression. These findings corroborate studies that analyzed the effects of stress at work and at home on employees’ mental and general health. It was found in previous studies that a significant cause of the increased perceived work-related stress by workers is the interference between work and private life. The authors identified that for men, high demands of work, insecurity, stress at work and home were the main aspects correlated with more symptoms of depression and anxiety^(25,26)^. This same correlation with symptoms was identified for job insecurity and stress at home for women. The social support, including the boss or co-workers, was negatively associated with worker anxiety, demonstrating a possible moderating effect. In this sense, the authors suggest that stress at work and the worker’s home, and social support should be considered together for an adequate analysis^(25)^.

The literature states that meditation practice is associated with decreased stress and improvement in various health conditions^(27)^. In the present study, meditation practice seems to suggest a protective effect against perceived stress. However, we did not investigate the type of meditation and the constancy of practices. In addition, it is relevant to consider the fact that we had a very small number of participants who reported meditating.

Stress is a silent disease that allows subjects to continue working even at harmful levels, and in most cases, the worker is only withdrawn or seeks help when he/she is ill or manifests symptoms of chronic stress in clinical conditions^(7)^.

The study findings are relevant to current occupational and environmental health practice because assessing perceived stress and relating the main predictors associated with this outcome may infer a better understanding of the phenomenon to plan future effective stress reduction interventions for workers. These measures can prevent illnesses resulting from chronic stress and improve the quality of life of this population.

Due to the worker’s risk of illness, dissatisfaction, and performance losses related to high levels of stress, the findings show that identifying the main associated factors is essential for occupational health nurses and other occupational health practitioners who work in universities and are responsible for helping workers. This knowledge allows the orientation and planning of further practical intervention actions, promoting mental health, and avoiding mental illness. From this perspective, the results of this study will be used to support worker health policies aimed at preventing, reducing, and controlling stress to improve workers’ quality of life in the context that this study was conducted.

The limitation in the present study is related to the low adherence (54.51%) of workers participating in the survey. Further studies could include a longitudinal analysis and cover more individuals to understand the phenomenon better.

## CONCLUSION

Our findings allowed us to identify the level of perceived stress in a sample of administrative technicians of a Brazilian public university and to determine the main predictors related to stress. We conclude that the predictors of perceived stress among workers in a higher education institution are related to being younger, male, occupying a higher level or technical position, and presenting higher scores of depression and anxiety.

Further research is required in other contexts to understand the perceived stress phenomenon better. The results obtained are relevant to understanding the main predictors of perceived stress to provide information to assist the occupational health nurse in managing employees’ health.
